# The Occurrence of Atopy in Patients with Isolated Spontaneous Mast Cell (or Nonallergic) Angioedema

**DOI:** 10.3390/jcm13020477

**Published:** 2024-01-15

**Authors:** Magdalena Zajac, Andrzej Bozek, Alicja Grzanka

**Affiliations:** Clinical Department of Internal Diseases, Dermatology and Allergology, Medical University of Silesia, 40-055 Katowice, Poland; magadalenazajac2000@gmail.com (M.Z.); agrzank@sum.edu.pl (A.G.)

**Keywords:** angioedema, IgE, allergic rhinitis, atopic dermatitis

## Abstract

Background: Isolated mast cell angioedema (MC-AE) can be divided into allergic and nonallergic (spontaneous) forms. The former is often associated with food, Hymenoptera venoms or drug allergies. This study aimed to evaluate the relationship between the occurrence of atopic diseases and the risk of angioedema. Methods: A retrospective study analyzed 304 patients with confirmed MC-AE and 1066 controls. All were analyzed for allergic asthma (AA), atopic dermatitis (AD) and allergic rhinitis (AR) based on ICD-10 codes. In addition, total IgE and peripheral eosinophilia were calculated. Results: The analyzed atopic diseases were more frequent in the group of patients diagnosed with MC-AE than in the controls: 78 (25.7%) vs. 173 (16.2%) for *p* < 0.01. Patients diagnosed with AD had a higher risk of MC-AE (hazard ratio (HR) = 1.48,) similar to those diagnosed with AR (HR = 1.51). However, in patients with two or three atopic comorbidities, the risk increased significantly to HR = 2.45 or HR = 4.1, respectively. There was a positive correlation between the serum total IgE concentration or eosinophilia and the risk of angioedema (*p* < 0.01). Conclusion: Patients with MC-AE had a more frequent occurrence of atopic diseases associated with inhalant allergies. This risk increased in patients with IgE-mediated polymorphic disease.

## 1. Introduction

Angioedema (AE) is the noninflammatory, self-limiting swelling of the deep layers of the skin, subcutaneous tissue and/or mucous membranes and submucosa caused by local vasodilation and increased permeability of the blood vessels due to the action of vasoactive mediators [1,2]. Isolated AE (without wheals) is classified into two classes: bradykinin AE (hereditary or acquired, with or without C1-INH deficiency) and allergic or spontaneous mast cell AE. AE most often affects adult patients, and histamine-mediated AE is the most common in this group [2,3]. Currently, it is classified as one of the forms of urticaria without wheals. Depending on the participation of individual mediators in the mechanism of angioedema, its forms are distinguished as those related to mast cell mediators (mainly histamine) and histamine-independent forms. The latter group includes primarily isolated angioedema (not associated with urticaria), especially their congenital forms, in which the mediator is mainly bradykinin [2].

The most common manifestation of isolated angioedema is limited asymmetrical edema of the skin and subcutaneous tissue. The skin covering the edematous lesions is not inflamed and does not itch. The swelling usually lasts from 48 to 72 h and is isolated or appears in several places. It most often affects the facial skin, mainly the lips and eyelids, but may also occur on the trunk, limbs and genital area. In some cases, the swelling also affects the mucous membranes of the respiratory tract; in this case, acute swelling of the throat and larynx may threaten the patient’s life. A rarer and often misdiagnosed manifestation of AE is changes in the gastrointestinal tract accompanied by pain, nausea, vomiting and diarrhea. Severe swelling in this location may imitate the symptoms of an “acute abdomen”. Symptoms affecting the urinary, musculoskeletal and central nervous systems may occur in particularly atypical forms of AE.

Despite attempts to associate angioedema with IgE-dependent allergies, especially by patients, these relationships are not very obvious. There is understandable interest in the relationship between angioedema and atopy. The common stereotype that angioedema is only a consequence of allergies has no simple scientific justification.

The genetically determined trait of atopy has a polygenic inheritance. The overproduction of IgE and the manifestation of allergic diseases are its main features.

Atopy refers to a highly allergenic state that can manifest in one or a constellation of the following: allergic asthma, atopic dermatitis and allergic rhinitis [4,5]. Patients with atopy are also predisposed to having food allergies, allergic conjunctivitis and other symptoms characterized by IgE-mediated allergic reactions [4,5].

This study aimed to assess the occurrence of atopy, understood as its clinical manifestation in the form of atopic diseases, allergic asthma, allergic rhinitis and/or atopic dermatitis, in patients diagnosed with isolated mast cell angioedema (MC-AE).

## 2. Methods

### 2.1. Study Design

**Figure 1:**
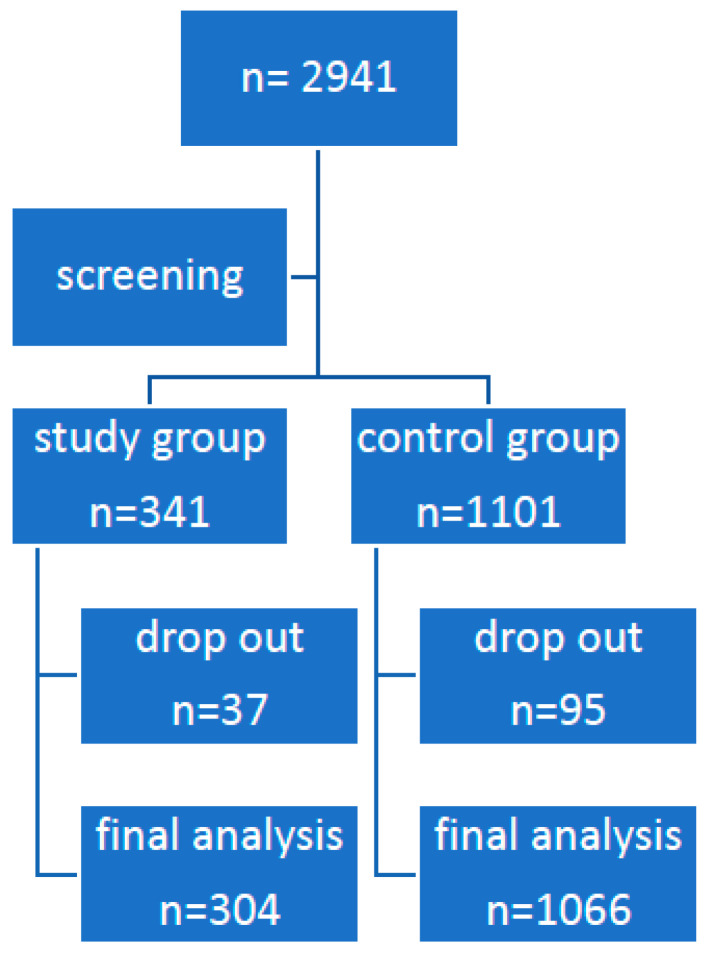
Flow diagram of the patient qualification process.

This study was retrospective and based on a group of people with a final diagnosis of angioedema (according to ICD code 10) in the last 10 years. People without a diagnosis of angioedema formed the control group. The study group included 304 patients, 145 women and 159 men, aged from 18 to 80, with a mean age of 49.3 ± 11.2 years. The group was selected by analyzing 2941 records (Figure 1).

The inclusion criteria for the study were as follows: age between 18 and 80 y and at least three documented isolated mast cell angioedema during 12 months of observation.

The exclusion criteria were as follows:

- angioedema with chronic urticaria;
- diagnosis of hereditary angioedema, which was also checked based on the ICD-10 codes, using the following medical history points: a positive family history; an onset of symptoms in childhood/adolescence; painful abdominal symptoms; the occurrence of upper airway edema; a lack of response to antihistamines, glucocorticoids or epinephrine; the presence of prodromal signs or symptoms before swelling; an absence of urticaria; a low level of C1-INH (antigen or function) or/and C4 in plasma; mutations in the SERPING1 gene and hereditary angioedema due to a mutation in factor XII; hereditary angioedema due to a mutation plasminogen; hereditary angioedema due to a mutation angiopoietin 1; or hereditary angioedema due to a mutation kininogen-1;
- mild angioedema-like symptoms, such as swelling of the tongue or mouth without visible edema;
- documented angioedema induced by drugs or the use of convertase inhibitors or their derivatives; however, patients for whom the discontinuation of convertase inhibitors or other suspected drugs did not resolve their edema symptoms were enrolled in the study;
- bradykinin forms (by C1-INH deficiency, by ACE inhibitor); nonallergic NSAID edema;
- any direct allergic forms of angioedema;
- reactions that could not be classified as angioedema, deficiencies in documentation and lack of consent.

Additionally, patients with a clinical diagnosis of hereditary angioedema were excluded from this study. A clinical diagnosis of hereditary angioedema was based on the following medical history points: a positive family history; an onset of symptoms in childhood/adolescence; painful abdominal symptoms; the occurrence of upper airway edema; a lack of response to antihistamines, glucocorticoids or epinephrine; the presence of prodromal signs or symptoms before swelling; and an absence of urticaria [1,6].

The control group was composed of 1066 people, including 488 women and 578 men aged 18–80 years, with an average age of 52.1 ± 13.8 years, selected from family doctor outpatient clinics. This group was matched in terms of age and sex to the patients in the study group. The characteristics of the patients are presented in Table 1.

The following procedures were carried out:

1. Assessment of the angioedema incident (ICD-10 code, medical history and documentation)
2. Analysis of the possibility of atopic diseases, including total and specific IgE to inhalant allergen results and diagnoses of atopic dermatitis, allergic asthma and allergic rhinitis.

The diagnoses of the listed atopic diseases were confirmed based on ICD-10 codes, medical history and treatment for at least one year.

Total serum IgE and serum-specific IgE (sIgE) reactivity to the inhalant allergens were determined using immunoenzymatic methods. The results were considered positive when the sIgE concentration was greater than 0.35 IU/mL. Additionally, peripheral blood eosinophilia and the concentration of C1-INH were analyzed.

The Bioethical Committee of the Medical University of Silesia approved the project in Katowice, Poland (no. KNW/0022/KB1/18/14).

### 2.2. Statistics

Statistica software 8.1 (StatSoft, Cracow, Poland) was used for calculations. The significance of differences for normal distributions was assessed with ANOVA and for nonnormal distributions with the Kruskal–Wallis test. The Spearman test was used to evaluate the correlation. Hazard ratios, 95% CIs and *p* values were calculated, and the model was adjusted for atopy, diagnosis of allergic asthma, allergic rhinitis and atopic dermatitis. *p* < 0.01 was considered statistically significant.

## 3. Results

The occurrence of any analyzed atopic diseases with high serum total IgE (>100 kU/L) levels was more frequent in the group of patients diagnosed with MC-AE than in the control group: 78 (25.7%) vs. 173 (16.2%) for *p* < 0.01. There was no correlation between the occurrence of allergic asthma and MC-AE events: hazard ratio (HR) = 1.17 (95% CI: 0.96–1.31). Patients diagnosed with AD had a slightly higher risk of AE, at HR = 1.48 (95% CI: 0.98–1.76), similar to those diagnosed with AR, at HR = 1.51 (95% CI: 1.12–1.82). However, in patients with two or three atopic comorbidities, the risk increased significantly to HR = 2.45 (95% CI: 2.21–3.12) or HR = 4.1, (95% CI: 3.72–4.43). No association of MC-AE with sensitization to the specific allergens tested was observed. However, patients allergic to three or more groups of allergens had an increased risk of MC-AE (HR = 2.98, 95% CI: 2.66–3.51).

### Laboratory Parameters

**Figure 2:**
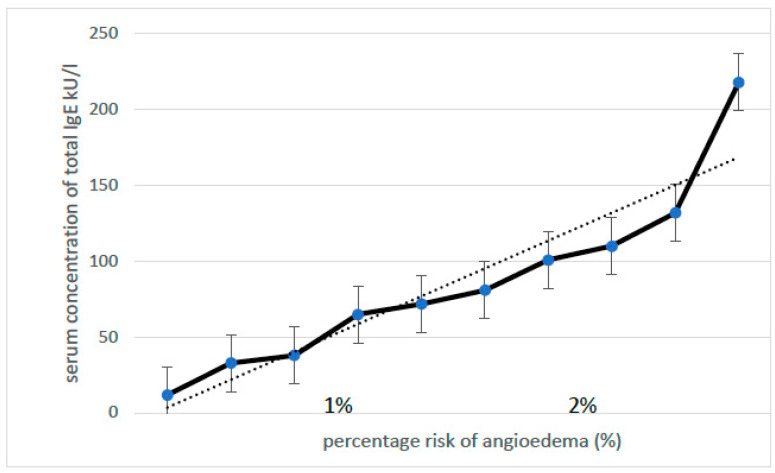
Correlation between the serum total IgE concentration and the percentage risk of angioedema. Legend: positive correlation between IgE and the risk of angioedema (Spearman test: r = 0.84, *p* < 0.01).

**Figure 3:**
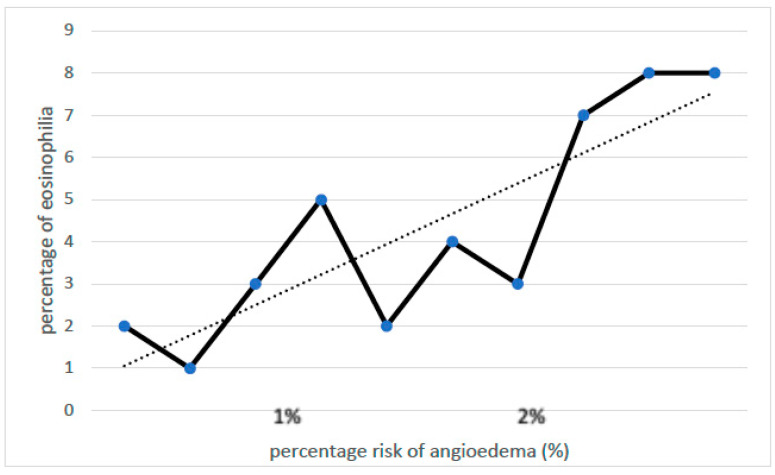
Correlation between the percentage of eosinophilia and the risk of angioedema. Legend: positive correlation between IgE and the risk of angioedema (Spearman test: r = 0.64, *p* < 0.01).

A relationship was observed between the increase in serum total IgE concentration and the frequency of MC-AE diagnosis (Figure 2). A less significant association was observed for the peripheral blood eosinophil count and MC-AE diagnosis (Figure 3).

## 4. Discussion

In everyday practice, the causes of MC-AE are not always assessed, despite recommendations [1]. Angioedema caused by mast cells—due to the release of histamines, among other substances—manifests itself in allergic reactions, urticaria or pseudo-allergic episodes. Unlike bradykinin-mediated angioedema, it is often accompanied by pruritus and/or hives/urticaria. Several studies have searched for the cause of angioedema [7,8,9,10,11]. For example, an association of angioedema with autoimmune reactions, e.g., in Hashimoto’s disease, has been observed by some authors. Similarly, the presence of selected neoplastic diseases, hypersensitivity to drugs (especially convertase inhibitors) and high levels of uric acid may favor the development of angioedema [12,13,14]. In these cases, autoimmune reactions are a common mechanism of the disease. However, most of these studies did not specify the types of angioedema observed apart from the obvious drug-related instances. However, it seems that angioedema mediated by bradykinin may be of primary importance there.

Isolated mast cell angioedema can also be divided into allergic and nonallergic forms. Allergy to foods, certain medications, and Hymenoptera venom are reported causes of this disease. IgE-dependent mast cell activation is the primary stimulus [2]. In allergic angioedema, mast cells liberate histamine, causing vasodilatation, itching and tissue edema formation. The release of mast cell cytokines stimulates a TH2 response with the influx of eosinophils, whose cytotoxic proteins cause sustained allergic inflammation [1,2,6]. There are no reports on the role of typical atopic diseases being associated with inhalant allergens.

In the present study, the influence of atopy was confirmed as another factor contributing to MC-AE. However, this does not rule out other types of immune responses, and further investigation is needed. Elevated serum total IgE values favored the occurrence of angioedema, which was confirmed in some studies [15]. In addition, a moderate relationship was observed between an increase in the peripheral blood eosinophil count and a higher incidence of angioedema. This is in opposition to the observations of another author [15]. A possible key connection is the presence of bradykinin as the primary mediator in most cases of acquired nonallergic angioedema and the accompanying IgE dependence of this disease, similar to histamine and PAF in the allergic form [2,16].

There are few studies on the relationship between the clinical manifestation of atopy and the tendency to develop angioedema without type specification. In one study, allergic asthma had no significant effect on the risk of edema [15]. However, allergic rhinitis, which seems to be the most “pure” atopic disease, increases this risk [15]. The most significant risk factor for angioedema was atopic multiform disease (e.g., asthma and allergic rhinitis or atopic dermatitis and allergic asthma), which is a novel finding that needs verification in larger study groups.

This study has some limitations. The relatively small study sample, lack of comparison with patients with chronic urticaria and the risk of coexisting non-IgE-dependent mechanisms in the induction of the studied angioedema may affect the final results. A more thorough analysis of inflammatory mediators in the study group would allow us to answer pending questions and explain the unclear findings. Currently, such a prospective study is planned.

## 5. Conclusions

Patients with isolated mast cell angioedema have a more frequent occurrence of atopic diseases associated with inhalant allergy. This risk increases in patients with IgE-mediated polymorphic disease. More research is needed in this area.

## Figures and Tables

**Table 1:** Characteristics of the study patients.

	Study Group *n* = 304	Control Group *n* = 1066	*p*
Mean age (±SD; in years)	49.3 ± 11.2	52.1 ± 13.8	NS
Mean BMI (kg/m^2^) ± SD	23.6 ± 2.1	25.1 ± 5.5	NS
Female (%)	145 (47.7)	488 (45.8)	NS
Rural area (%)	122 (31.9)	356 (33.3)	NS
Present or former smokers (%)	91 (29.9)	341 (33)	NS
Allergic asthma	35 (11.5)	101 (9.5)	0.05
Allergic rhinitis	104 (34)	281 (26.3)	<0.01
Atopic dermatitis	14 (4.6)	21 (2)	<0.01
Allergy to			
house dust mites	28 (9.2)	77 (7.2)	NS
Grass pollen	26 (8.6)	54 (5)	<0.01
Tree pollen	21 (7)	55 (5.2)	NS
Animal dander	9 (3)	19 (1.8)	NS
Molds	6 (2)	13 (1.2)	NS
Localization of AE		-	-
Mouth	189 (62)		
Eye area	124 (40.8)		
Throat	34 (11.2)		
Hands or feet	86 (28)		
Others	19 (8)		

Legend: AE—angioedema; BMI—body mass index; SD—standard deviation; NS—not significant.

## Data Availability

The data presented in this study are available on request from the corresponding author. The data are not publicly available due to ethical restrictions.
